# A novel non-canonical Wnt signature for prostate cancer aggressiveness

**DOI:** 10.18632/oncotarget.14161

**Published:** 2016-12-24

**Authors:** Elise Sandsmark, Ailin Falkmo Hansen, Kirsten M. Selnæs, Helena Bertilsson, Anna M. Bofin, Alan J. Wright, Trond Viset, Elin Richardsen, Finn Drabløs, Tone F. Bathen, May-Britt Tessem, Morten B. Rye

**Affiliations:** ^1^ Department of Circulation and Medical Imaging, Faculty of Medicine, NTNU - Norwegian University of Science and Technology, Trondheim, Norway; ^2^ Department of Urology, St. Olavs Hospital, Trondheim University Hospital, Norway; ^3^ Department of Cancer Research and Molecular Medicine, Faculty of Medicine, NTNU - Norwegian University of Science and Technology, Trondheim, Norway; ^4^ Department of Laboratory Medicine, Children's and Women's Health, Faculty of Medicine, NTNU - Norwegian University of Science and Technology, Trondheim, Norway; ^5^ Cancer Research UK Cambridge Institute, University of Cambridge, United Kingdom; ^6^ Department of Pathology and Medical Genetics, St. Olavs Hospital, Trondheim University Hospital, Norway; ^7^ Department of Medical Biology, UiT - The Arctic University of Norway, Tromsø, Norway; ^8^ Department of Clinical Pathology, University Hospital of North Norway, Tromsø, Norway; ^9^ St. Olavs Hospital, Trondheim University Hospital, Norway

**Keywords:** EMT, gene expression signature, biochemical recurrence, spectroscopy, MRSI

## Abstract

Activation of the Canonical Wnt pathway (CWP) has been linked to advanced and metastatic prostate cancer, whereas the Wnt5a-induced non-canonical Wnt pathway (NCWP) has been associated with both good and poor prognosis. A newly discovered NCWP, Wnt5/Fzd2, has been shown to induce epithelial-to-mesenchymal transition (EMT) in cancers, but has not been investigated in prostate cancer. The aim of this study was to investigate if the CWP and NCWP, in combination with EMT, are associated with metabolic alterations, aggressive disease and biochemical recurrence in prostate cancer. An initial analysis was performed using integrated transcriptomics, *ex vivo* and *in vivo* metabolomics, and histopathology of prostatectomy samples (n=129), combined with at least five-year follow-up. This analysis detected increased activation of NCWP through Wnt5a/ Fzd2 as the most common mode of Wnt activation in prostate cancer. This activation was associated with increased expression of EMT markers and higher Gleason score. The transcriptional association between NCWP and EMT was confirmed in five other publicly available patient cohorts (1519 samples in total). A novel gene expression signature of concordant activation of NCWP and EMT (NCWP-EMT) was developed, and this signature was significantly associated with metastasis and shown to be a significant predictor of biochemical recurrence. The NCWP-EMT signature was also associated with decreased concentrations of the metabolites citrate and spermine, which have previously been linked to aggressive prostate cancer. Our results demonstrate the importance of NCWP and EMT in prostate cancer aggressiveness, suggest a novel gene signature for improved risk stratification, and give new molecular insight.

## INTRODUCTION

Increased activation of the Wnt signaling pathway (WP) is associated with development, progression, and metastasis of many cancers [1]. In prostate cancer, the WP has been associated with aggressive, late stage disease, and metastasis [2–5]; however, its potential for early prediction of aggressiveness is still unclear. Previous studies are mainly performed in prostate cancer cell lines [6–9], and proper validation in human tissue is lacking. The WP is proposed as a therapeutic target in prostate cancer treatment [10], and reduced proliferation has been detected as a result of targeted Wnt-inhibitor drugs in cell lines [11, 12]. However, to develop Wnt-targeted drugs for human prostate cancer, an increased understanding of the molecular mechanisms *in vivo* is needed.

**Figure 1:**
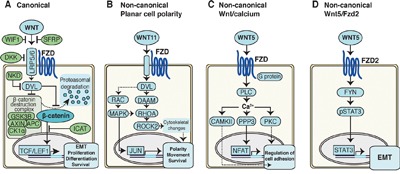
Schematics of Wnt signaling pathways in cancer cells **A**. Canonical Wnt pathway. In the absence of Wnt signaling, the β-catenin destruction complex labels β-catenin for proteasomal degradation. In the presence of Wnt signaling, the destruction complex is inhibited, resulting in stabilization and nuclear translocation of β-catenin, activating transcription of target genes. **B**. Non-canonical planar cell polarity (PCP) pathway activates signaling cascades resulting in cytoskeletal changes, as well as alterations in cell polarity, movement and survival. **C**. Non-canonical Wnt/Calcium pathway signaling activates intracellular calcium, which in turn reduce cell adhesion through further signaling. **D**. Non-canonical Wnt5/Fzd2 pathway. Wnt5 signals via the FZD2 receptor and FYN activates STAT3 transcription leading to epithelial-mesenchymal transition (EMT) in cancer cells.

Wnt ligands bind to Frizzled (Fzd) receptors to activate the WP, which then induces signal transduction cascades. The WP is generally divided into a β-catenin-dependent canonical WP (CWP), and a β-catenin-independent non-canonical WP (NCWP). The importance of the CWP in carcinogenesis was first discovered in colorectal cancer, where mutations of the *APC* gene, a part of the β-catenin destruction complex (Figure 1A), resulted in stabilization and nuclear translocation of β-catenin [13]. This β-catenin translocation is a hallmark of CWP activation, and can drive tumor invasion and metastasis through a process of epithelial-to-mesenchymal transition (EMT) [14]. During EMT, epithelial cancer cells develop into less adhesive and more motile mesenchymal-like cells, which increases the cancer's potential for invasion and metastasis [15]. There is mounting evidence associating EMT in prostate cancer with increased aggressiveness [16]. Several studies support the activation of CWP in advanced and metastatic prostate cancer [7, 17], but little evidence exists for localized and locally advanced prostate cancer.

The NCWP is commonly divided into two pathways, the planar cell polarity (PCP), and the Wnt/Calcium pathway (Figure 1B-1C). Few studies have addressed the significance of NCWP in prostate cancer. Most attention has been focused on the role of the non-canonical ligand Wnt5a, a key activator of the NCWP. Wnt5a is generally found to be upregulated in prostate cancer, but results are inconsistent regarding its association with good [18–20] or poor prognosis [21]. Recently, a new NCWP involving Wnt5a and the receptor Frizzled2 (Fzd2) was discovered (Figure 1D) and shown to promote tumor progression and EMT in several cancer cell lines and a mouse xenograft model [22]. In the same study, a Wnt5/Fzd2 based gene set was also shown to accurately predict metastasis and survival in a small cohort (n=46) of patients with hepatocellular carcinoma. However, this study did not address the *in vivo* relevance of the NCWP in larger patient cohorts or in prostate cancer tissue.

Metabolic reprogramming is a hallmark of cancer [23], and the WP has been suggested as an emerging mediator of cancer cell metabolism [24, 25]. Wnt5a-mediated NCWP has been directly related to alterations of the energy metabolism in melanoma and breast cancer cells [26]. Selected metabolic alterations detected in tissue samples by high resolution magic angle spinning magnetic resonance spectroscopy (HR-MAS MRS) can be translated for use in a clinical setting by magnetic resonance spectroscopy imaging (MRSI). Differences in (choline + creatine + spermine)/citrate ratio between low and high histopathological Gleason score have previously been detected using *in vivo* MRSI of patients [27], and citrate and spermine are suggested as the main contributors to discriminating on the basis of tumor aggressiveness from tissue HR-MAS MRS analysis [28]. To date, metabolic alterations associated with the WP have not been investigated in prostate cancer.

The aim of this study was to investigate if the CWP and NCWP, in combination with EMT markers, are activated and associated with aggressive disease and metabolic alterations in human prostate cancer. To approach these questions, we first used a patient cohort where integrated omics analyses were performed on the same samples from fresh-frozen prostatectomy-tissue slices, including transcriptomics, tissue *ex vivo* and *in vivo* patient metabolomics, and detailed histopathological evaluation [29]. Histopathology allowed us to control for tissue heterogeneity, particularly the fraction of stroma, which is a major complicating factor when analyzing tissue samples [30]. The findings were confirmed in publicly available prostate cancer cohorts (n=1519 samples in total), and in a separate immunohistochemistry cohort. The analysis suggests that the NCWP, and not the CWP, is the most active WP for *in vivo* prostate cancer, and that this activity correlates with markers for EMT. Our approach allowed for the development of a novel NCWP-EMT gene signature significantly associated with recurrent and metastatic cancer and metabolic biomarkers. This signature may help differentiate aggressive from indolent prostate cancer.

## RESULTS AND DISCUSSION

Patient and sample characteristics of the *main* and the *immunohistochemistry cohorts* are presented in Table 1. The five *validation cohorts* (in total 1519 samples) are presented in the methods section with more information listed in Supplementary Table 1.

**Table 1:** Patients and sample characteristics of the two cohorts

		Main cohort	Immunohistochemistry cohort
Patients		n=41	n=40
Age (median, range)	Years	64 (48-69)	62 (48-73)
sPSA (median, range)	Before Surgery (ng/mL)	9.1 (4.0-45.8)	8.9 (5.2-18.0)
Clinical pT stage (patients)	pT1c	-	7
	pT2	28	20
	pT3	13	10
	Unknown	-	3
**Tissue samples**		**n=129**	**n=40**
Sample weight (mean, range)	(mg)	12.7 (3.0-21.9)	12.6 (7.6-21.0)
	Benign	34	- *
Gleason score of tissue samples	6	24	5
	7	41	25
	8	15	5
	9	15	4
	10	-	1
**Gleason grade groups**	*Low Gleason (≤3+4)*	48	21
	*High Gleason (≥4+3)*	47	19

*50 benign samples were excluded from further analysis in the *immunohistochemistry cohort*

sPSA – serum PSA, pT stage – pathological tumor stage.

### The canonical Wnt pathway is not activated in prostate cancer

**Figure 2:**
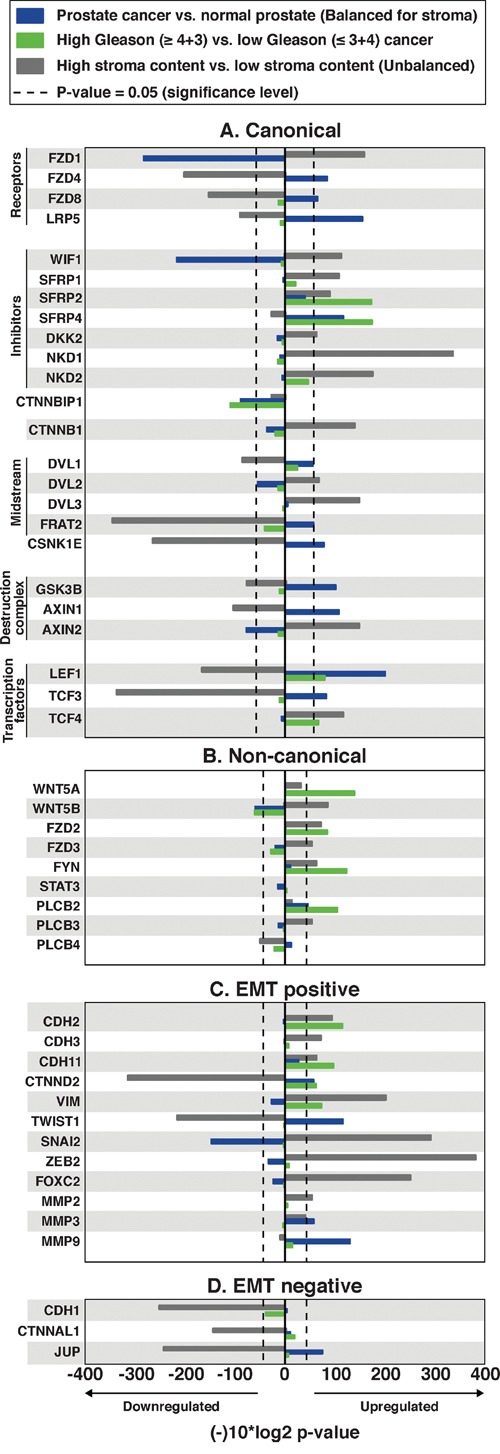
Alterations in central Wnt and EMT genes in prostate cancer compared with normal samples (balanced for stroma), *high Gleason* compared with *low Gleason* prostate cancer, and high stroma content compared with low stroma content (unbalanced) tissue samples The x-axis displays log10(p-value) fold change, multiplied by −1 for upregulated genes, and 1 for downregulated genes. P-values for prostate cancer vs. normal prostate tissue are balanced for stroma content; unbalanced p-values are available in Supplementary Table 2. **A**. The central canonical genes show a pattern of no further activation in cancer or *high Gleason* cancer, but show a confounding stroma effect, especially of the genes of the destruction complex. **B**. The central non-canonical genes generally show an upregulation of Wnt5/Fzd2 genes in *high Gleason* cancer. **C**. The central epithelial-mesenchymal transition (EMT) positive genes indicate ongoing EMT, especially in *high Gleason* cancer. **D**. The central EMT negative genes.

To investigate if the CWP is activated in prostate cancer, we compared gene expression of the central CWP genes between cancer and normal samples of the *main cohort* using sample subsets balanced and unbalanced for stroma content according to histopathology (Figure 2A, Supplementary Table 2, Methods). The level of β-catenin (*CTNNB1*), the key component of the CWP pathway, showed no significant altered expression in cancer compared to normal, and two of the main components of the β-catenin destruction complex, *GSK3B* and *AXIN1*, were significantly upregulated in cancer. This may suggest increased activity of β-catenin destruction in prostate cancer, contrary to what is expected when the CWP is turned on. Additionally, the Wnt ligand genes associated with the CWP were not significantly changed in cancer compared to normal samples. Other important findings are reduced expression of the receptor *FZD1*, increased expressions of the antagonist *SFRP4* and casein kinase *CSNK1E*, which support the absence of CWP activation. Although some variations were observed (Figure 2A), the lack of upregulation of the main CWP genes suggests no increased expression activity of the CWP in prostate cancer in our *main cohort*.

**Figure 3:**
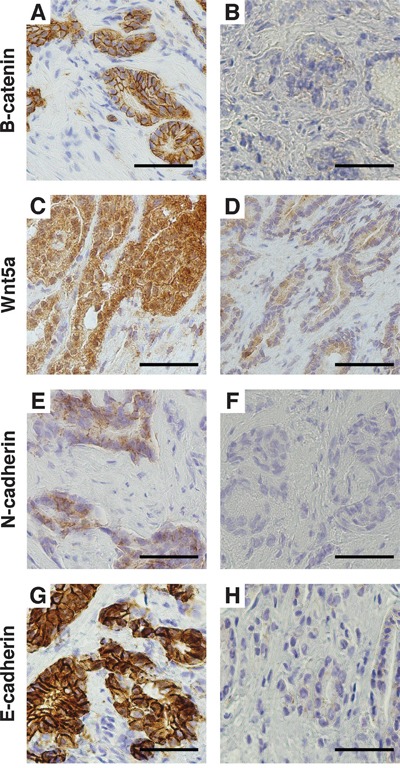
Immunohistochemical staining of the *immunohistochemistry cohort* **A**. Strong membranous β-catenin staining and **B**. weak β-catenin staining. **C**. Strong Wnt5a staining and **D**. weak Wnt5a staining. **E**. Positive membranous N-cadherin staining and **F**. negative N-cadherin staining. **G**. Strong membranous E-cadherin staining and **H**. weak E-cadherin staining. Magnification x400. Bar 50μm.

Translocation of β-catenin from the membrane to the nucleus is the hallmark of CWP activation, and to validate the findings above, we performed β-catenin immunohistochemistry (IHC) on the *immunohistochemistry cohort* (Figure 3A-3B). All the samples (n=40) had weak or non-detectable nuclear staining (SI≤2). Most of the samples (n=30) had strong membranous β-catenin staining (SI=9), indicating no activation of the CWP. Ten samples had weak or moderate membranous staining (SI≤6), indicating reduced membranous expression without increased nuclear expression of β-catenin. These findings demonstrate that the CWP is not activated in prostate cancer in our *immunohistochemistry cohort*, which is in concordance with the gene expression results from the *main cohort*. We therefore conclude that there is little evidence of CWP activation in prostate cancer compared to normal prostate tissue investigated in two independent cohorts.

We further investigated alterations in the CWP between *low Gleason (≤3+4)* and *high Gleason (≥4+3)* samples (Figure 2A). There were no significant gene expression alterations detected for β-catenin (*CTNNB1*), the Wnt ligands, the receptor-complex and the destruction complex (Supplementary Table 2). Of the CWP inhibitors, both *SFRP2* and *SFRP4* were upregulated in *high Gleason* compared to *low Gleason* cancer samples, which is contradictory to CWP activation. However, the inhibitor of β-catenin translocation, *ICAT* (*CTNNBIP1*), was downregulated, and the CWP transcription factors *LEF1* and *TCF* were upregulated in *high Gleason* cancer, which could indicate activation of downstream components of the pathway independently of the β-catenin destruction complex. To conclude, the overall analysis suggests no significant increase in CWP activation through the canonical destruction complex, neither in cancer compared to normal nor in *high Gleason* cancer.

There is currently no consensus in the literature regarding CWP activation in prostate cancer, and our findings are contradictory to several previous studies suggesting increased CWP in prostate cancer [7, 9, 17]. The CWP has previously been associated with advanced disease such as androgen resistant prostate cancer in cell lines [7], and prostate cancer bone metastasis in human tissue and cell lines [8, 17]. The fact that our cohorts consist of radical prostatectomy tissue, from localized or locally advanced disease, may explain the absence of CWP activation. The CWP may therefore still be of importance in advanced, metastatic prostate cancer, but might not prove useful for early risk stratification. Furthermore, several previous studies reporting increased CWP signaling were using prostate cancer cell lines [6–9]. The disparity could therefore also highlight a difference between *in vitro* cell lines and human prostate tissue, emphasizing the importance of validation studies in human tissue, especially for identification of potential targets for personalized drug therapy.

In our *main cohort*, the central CWP genes showed an expression pattern that was indicative of substantial stromal influence when comparing normal against cancer tissue (Figure 2A). This trend was particularly strong for genes that, directly or partly, regulate the activity of the β-catenin destruction complex, and indicates a difference of CWP activity when cancer is compared to stroma, but not when compared to benign epithelium. Thus, at least some of the discrepancies from previous studies of CWP in prostate cancer may be explained by uneven sampling of stroma content between cancer and normal samples which has previously been observed in tissue samples from prostate cancer patient cohorts [30, 31].

### Wnt5a-induced non-canonical Wnt signaling is increased in *high Gleason* prostate cancer

The NCWP, including the Wnt/Calcium, PCP and the new Wnt5/Fzd2 pathways, were investigated (Figure 2B, Supplementary Table 2). When comparing cancer with normal samples, we found no alterations in any of the pathway components apart from downregulation of the ligand *WNT5B*, and upregulation of the calcium pathway component *PLCB2*, suggesting no increased activation of the NCWP in prostate cancer in general. However, when *high Gleason* samples were compared with *low Gleason* samples, significantly increased expressions were detected for three of the four key genes of the Wnt5/Fzd2 pathway; the ligand *WNT5A* (p<0.001), the receptor *FZD2* (p=0.003) and the midstream kinase component *FYN* (p<0.001) (Figure 2B). No significant expression change was detected for the last key component, the transcription factor *STAT3*. For the Wnt/Calcium pathway, only *PLCB2* was upregulated in *high Gleason* cancer (Figure 2B), and none of the central components of the PCP pathway were altered (Supplementary Table 2). In summary, these data suggest upregulation of the Wnt5/Fzd2 pathway in *high Gleason* prostate cancer.

For validation, IHC of WNT5A was performed on the *immunohistochemistry cohort* (Figure 3C-3D). Of the 40 cancer samples, 32 had strong (SI=9) and 8 had moderate or weak staining (SI≤6). There was no association between the staining intensity and Gleason grade for this cohort.

Wnt5a has been suggested as a biomarker in prostate cancer, but its prognostic outcome has been inconsistent [18–21]. The increased *WNT5A* gene expression in *high Gleason* cancer samples compared to *low Gleason* samples is in agreement with results from Yamamoto et al. who reported increased Wnt5a IHC staining of prostatectomy tissue samples with high Gleason grade [21]. This oncogenic effect of Wnt5a in prostate cancer progression is also supported by studies of cell lines, where Wnt5a has been shown to improve migration capacity [32], induce androgen resistance in prostate cancer metastases [33], and induce bone metastasis [8]. Contrary to this, other IHC studies of prostatectomy tissue samples have detected a tumor-suppressing role of Wnt5a in prostate cancer; increased Wnt5a IHC expression has been associated with increased 10 years survival [18], and a lower risk of biochemical recurrence [19, 20]. This was, however, only true for low Gleason grade samples in one of the studies [20]. This apparent opposing role of Wnt5a in prostate cancer may be explained by the paradoxical effect of Wnt5a in other cancers. In melanoma, pancreatic and gastric cancer, Wnt5a expression is associated with worse prognosis, but in colon and thyroid cancer Wnt5a expression is associated with better prognosis as reviewed by McDonald and Silver, and Zhu et al. [34, 35]. The tumor-promoting role of Wnt5a can be caused by activation of NCWP [35], whereas the tumor-suppressing role may be caused by inhibition of the CWP [36]. Because of this conflicting role in different cancer types, we suspect that Wnt5a alone may not be a useful biomarker for prostate cancer.

### EMT markers are upregulated in *high Gleason* prostate cancer

**Figure 4:**
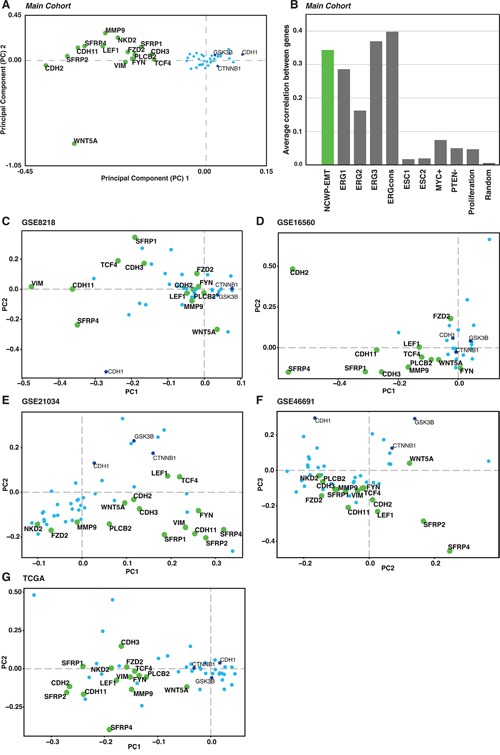
The NCWP-EMT gene expression signature **A**. Two component PCA plot reveals a group of 15 of 48 genes, mainly connected to Wnt5a/Fzd2 non-canonical Wnt pathway, epithelial-mesenchymal transition (EMT), and inhibitors of the canonical Wnt pathway, collectively termed NCWP-EMT (*CDH2, CDH3, CDH11, FYN, FZD2, LEF1, MMP9, NKD2, PLCB2, SFRP1, SFRP2, SFRP4, VIM, TCF4 WNT5A*). **B**. The Pearson correlation of co-expression of the genes in the NCWP-EMT signature is as good or better compared with other recognized genes expression signatures in prostate cancer. Random marks 200 randomly selected genes for validation. **C-G**. The NCWP-EMT signature confirmed in the *validation cohorts*, although there were some variations in the highlighted genes. High-resolution versions of the PCA plots including all gene names, and Pearson correlation of the *validation cohorts* are available in Supplementary Figure 1.

The Wnt5/Fzd2 NCWP has previously been linked with EMT studies on various cancer cell-lines, but not in prostate cancer [22]. We therefore evaluated the gene expression of the most central EMT positive and negative markers in prostate cancer in the *main cohort* (Figure 2C and 2D). When comparing *high Gleason* with *low Gleason* samples, significant upregulations were detected for the expression of EMT positive markers in *high Gleason*; N-cadherin (*CDH2*), OB-cadherin (*CDH11*), vimentin (*VIM*) and Delta-2-catenin (*CTNND2*) (Figure 2C). In addition, a non-significant downregulation of E-cadherin (C*DH1*), an EMT negative marker, was observed in *high Gleason* samples (fold-change=-0.25, p=0.07; Figure 2D), suggesting ongoing EMT in *high Gleason* samples. In the *immunohistochemistry cohort*, IHC of N-cadherin showed membranous staining (SI≥2) in only two, both *high Gleason*, of the forty cancer samples (Figure 3E-3F). Reduced, moderate membranous staining of E-cadherin (SI=6), was detected in five samples while the remaining samples had strong membranous staining (SI=9) (Figure 3G-3H). However, the reduced E-cadherin staining did not correspond to N-cadherin staining, as hypothesized for the N- to E-cadherin switch proposed to be important for EMT in prostate cancer [37]. Inspection of the principal component analysis (PCA) score plots for the *main* and *validation* cohorts also confirmed consistent N-cadherin upregulation correlating with *high Gleason* and EMT genes, while the anticorrelation to E-cadherin was inconsistent between the cohorts, in accordance with observations in the *immunohistochemistry cohort* (Figure 4A, 4C-4G). In conclusion, the increased levels of several EMT positive genes, suggests ongoing EMT in a subset of mainly *high Gleason* prostate cancer samples. This was partly supported by the IHC, although the number of samples in the *immunohistochemistry cohort* was too few to make a conclusion.

### A novel 15 gene non-canonical Wnt pathway - EMT (NCWP-EMT) signature

To further investigate the relationship between the expression of Wnt and EMT genes, PCA analysis was performed on the expression profiles of 48 central Wnt and EMT genes (Methods). The first two principal components clearly highlighted a separate cluster of 15 genes related to the Wnt5a/Fzd2 pathway and EMT (Figure 4A). This gene set included 11 genes, which were also upregulated in *high Gleason* samples. In addition, two inhibitors of the CWP (*NKD2* and *SFRP1*), and two EMT positive markers (*CDH3* and *MMP9*) were part of the PCA cluster and included in the gene set. Because of the clear relationship to Wnt5/Fzd2 NCWP and EMT, we will refer to this set of genes collectively as the NCWP-EMT genes.

Using all cancer samples in the *main cohort*, we calculated an average Pearson's correlation r of 0.34 between all 15 gene using pairwise correlations. This is comparable or higher than the average correlation between genes in previously validated prostate cancer signatures [38, 39] (Figure 4B), including signatures for the established TMPRSS2-ERG gene fusion (average Pearson's r=0.30). The pattern of the NCWP-EMT gene set from the *main cohort* was validated in PCA analysis of the Wnt-genes in the five publicly available cohorts (n=1519 samples in total, Supplementary Table 1). The same 48 central Wnt-genes, in addition to WNT1, WNT3 and WNT3A which were lacking data in the *main cohort, were used*. All cohorts confirm the NCWP-EMT component as the most important source of variation in the gene expression, although there were some variations in the highlighted genes (Figure 4C-4G and Supplementary Figure 1). The CWP was either insignificant or spanning a separate axis of variation with little correlation to EMT. Interestingly, *WNT5A* expression pattern varied considerably with respect to the NCWP-EMT axis. Overall, these data show the NCWP-EMT gene cluster to be robust over large prostate cancer patient cohorts, and the 15 NCWP-EMT genes to be accessible for a concordant NCWP-EMT gene expression signature.

The continuous single sample gene set enrichment analysis (GSEA) score of the novel NCWP-EMT signature was significantly correlated with the Gleason score of the samples (Pearson's r of 0.49, p<0.001). When the samples were categorized according to the NCWP-EMT score as *low*, *intermediate*, and *high*, the distribution of *low/high Gleason* samples in the groups were as following: NCWP-EMT *low* (n=25/n=7), NCWP-EMT *intermediate* (n=17/n=14), and NCWP-EMT *high* (n=6/n=26). As expected most samples with *high* NCWP-EMT score also were *high Gleason* samples; however, some samples were *low Gleason*, and vice versa for samples with *low* NCWP-EMT score. This indicates that the NCWP-EMT signature might add an additional dimension for stratification, compared to Gleason grade alone. The NCWP-EMT signature may therefore, with further refinements and validation, be a useful addition to the selection criteria for active surveillance in prostate cancer patients.

The novel NCWP-EMT signature also showed significant association with previously published mesenchyme and cytokine gene signatures (Supplementary Figure 2), and highly significant gene ontology (GO) terms related to cell adhesion, extracellular matrix, inflammation and immune response which are features commonly associated with EMT (Supplementary Table 3). The same analysis based on the expression level of *WNT5A* alone, did not produce any significant GO terms, further supporting the hypothesis that Wnt5a alone is an ambiguous biomarker in prostate cancer.

### The NCWP-EMT gene signature is associated with metabolic alterations

We further investigated the metabolic alterations of 23 metabolites between samples with *low*, *intermediate*, and *high* activation of the developed NCWP-EMT gene expression signature (Supplementary Table 4) in the *main cohort*. The most prominent alterations were observed for the metabolites citrate and the polyamine spermine (Table 2), which showed significantly decreased concentration in the *high* NCWP-EMT compared to *low* NCWP-EMT samples. This alteration was also observed for *high* NCWP-EMT samples when compared with *intermediate* NCWP-EMT samples, but not when comparing *intermediate* with *low* NCWP-EMT samples. This suggests citrate and spermine alterations to be more profound in the samples with *high* NCWP-EMT score compared to *low* and *intermediate* score NCWP-EMT. In addition, there were alterations in the concentration of phosphoethanolamine and taurine between the *low* and the *intermediate score* group (p=0.002, p=0.028 respectively).

**Table 2:** Alterations in citrate and spermine metabolism

	Metabolite concentration (mmol/kg wet weight) *ex vivo* and metabolites amount/ratios *in vivo*			p-values^a^		
Signature score	*Low*	*Intermediate(Int)*	*High*	*Low* vs. *High*	*Int*. vs *High*	*Low* vs. *Int*.
	Median (IQR)	Median (IQR)	Median (IQR)			
***Ex vivo*** (n=95)	(n=32)	(n=31)	(n=32)			
Citrate	7.31 (5.57-11.56)	6.38 (4.56-11.58)	3.55 (2.08-7.25)	3.38·10^-4^*	0.018*	0.282
Spermine	1.55 (1.02-2.36)	1.23 (0.67-2.27)	0.75 (0.39-1.43)	3.38·10^-4^*	0.028*	0.113
						
***In vivo*** (n=22)	(n=10)	(n=7)	(n=5)			
Citrate/Creatine	7.36 (5.81-8.79)	4.45 (3.34-7.79)	2.77 (1.48-3.00)	0.0056*	0.027*	0.030*
Spermine/Creatine	0.83 (0.44-1.04)	0.50 (0.04-1.11)	0.00 (0.00-0.02)	0.0057*	0.027*	0.101

IQR – Interquartile range

* Indicates significance at p<0.05

^a^ P-values from LMM adjusting for multiple samples per patient, and corrected for multiple testing by Benjamini and Hochberg procedure.

Decreased concentrations of citrate and spermine have been associated with aggressive prostate cancer [28, 40], and our results therefore suggest the NCWP-EMT signature to be associated with an aggressive metabolic profile. Reduced citrate can be a result of increased energy production through the Krebs cycle in prostate cancer [41]. Previously, Wnt5a signaling has been identified as a regulator of the energy metabolism in melanoma cancer cells [26], and alterations of this metabolism have also been associated with EMT in cancer [42]. Another study detected that reduced polyamine content promoted EMT in non-tumor MDCK cells [43]. We therefore hypothesize that NCWP-EMT activation is associated with alterations in citrate and spermine metabolism in prostate cancer, although the direct mechanisms require further investigation.

To investigate the potential clinical translation of the metabolic findings, we inspected the gene signature score with matched pre-surgical *in vivo* MRSI from the same patients. Reduced citrate/creatine and spermine/creatine ratios were detected for *high* NCWP-EMT score samples when compared with *low* NCWP-EMT score (Table 2). Although we had a limited number of matched samples in the *main cohort* (n=22), the results support our findings from the tissue analysis, and demonstrates that the MR biomarkers can reflect the NCWP-EMT signature also in non-invasive MRSI examinations.

Citrate and spermine are stored within the luminal space of the glands in prostate tissue, and the observed metabolic alterations can be due to cell metabolism or morphological changes. In the *main cohort*, the citrate and spermine concentrations were correlated with luminal space (Spearman's rho=0.30/p=0.003, rho=0.31/p=0.003, respectively). This was a weaker correlation than between citrate and spermine concentrations and the NCWP-EMT signature score (Spearman's rho=0.42/p<0.001, rho=0.38/p<0.001, respectively). LMM, adjusting for luminal space as well as other tissue heterogeneity and Gleason score, still showed the same metabolic alterations to be significant (Supplementary Table 5). These results suggest the alterations observed in citrate and spermine concentrations are a combination of changes in both luminal space and reprogramming of metabolism in samples with *high* NCWP-EMT score. There was no relationship between Wnt5a expression and metabolite concentrations in either the *main* or *immunohistochemistry cohort* (Supplementary Table 6). This supports that Wnt5a should be used as a biomarker in combination with other pathway components, such as our NCWP-EMT signature.

### NCWP-EMT signature may help predict biochemical recurrence

**Figure 5:**
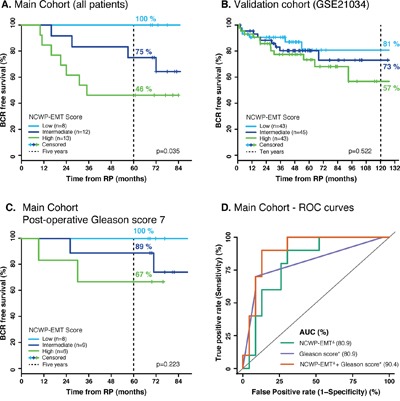
Kaplan-Meier and ROC curves of biochemical recurrence **A**. The *main cohort* shows clear separation in biochemical recurrence free survival between the *low*, *intermediate* and *high* NCWP-EMT signature groups. **B**. A validation cohort (GSE21034) shows the same pattern, although not a significant separation. **C**. A similar pattern was also shown for the patient of the *main cohort* with a post-operative Gleason score of 7. **D**. The ROC curves of biochemical recurrence after 5 years show the same AUC of post-operative Gleason score and NCWP-EMT, but an increased AUC when combined. ^Δ^ Continuous NCWP-EMT signature score, * continuous post-operative Gleason score. Abbreviations: BCR - biochemical recurrence, RP – radical prostatectomy, ROC – Receiver operating characteristic, and AUC – area under the curve.

In the *main cohort* the five-year biochemical recurrence free rates were 100%, 75% and 46% for the patients in the *low*, *intermediate* and *high* NCWP-EMT score groups, respectively, and the Kaplan-Meier plot showed a significant separation between the groups (log-rank p=0.035) (Figure 5A). Validation of recurrence was possible in the GSE21034 cohort (131 samples, 27 with recurrence), and showed a similar pattern with 10-year biochemical recurrence free rates of 81%, 73% and 57% in patients with *low, intermediate* and *high* NCWP-EMT score, respectively. However, there was no significant separation in the Kaplan-Meier curves for this cohort (log-rank p=0.522) (Figure 5B). For this validation dataset there was only one sample per patient, not necessarily extracted from the most aggressive cancer foci, which may reduce the precision of the NCWP-EMT grouping for biochemical recurrence analysis. In addition, many of the patients in the validation dataset were lost to follow-up early, and therefore censored in the analysis (Figure 5B), causing reduces reliability of the curves. In the GSE46691 cohort, samples with *high* NCWP-EMT scores were significantly associated with metastases (545 samples, 212 with metastasis, p-value<0.001, chi-square test, Supplementary Figure 3). With the significant separation in our data, and the similar trend in the validation datasets, we therefore suggest that increased NCWP-EMT signature score is associated with an increased risk of biochemical recurrence and metastases. This strengthens the NCWP-EMT signature, and the activation of the Wnt5/Fzd2 pathway, as markers of aggressive prostate cancer.

Patients in the *main cohort* with a post-operative Gleason score of 7 showed a five-year biochemical recurrence free survival of 100%, 89% and 67% with *low*, *intermediate* and *high* NCWP-EMT score, respectively (Figure 5C). Although not statistically significant, possibly due to the low number of patients (n=23), this separation with no crossing indicates that the NCWP-EMT gene signature might be useful for improved risk stratification in the challenging group of patients with Gleason score 7.

Univariate cox proportional hazards analyses identified NCWP-EMT, Gleason score and pathological T-stage as significant predictors of biochemical recurrence (Table 3). Multivariate analysis showed both NCWP-EMT and post-operative Gleason score to be significant predictors of biochemical recurrence (Table 3). The multivariate model included a significant interaction term between NCWP-EMT and post-operative Gleason score, implying that the hazards ratio of these variables were dependent on the value of the other variable. For patient with low post-operative Gleason score (≤ 7), the hazard ratio for NCWP-EMT was 1.61, indicating that increased NCWP-EMT signature score gives a significant higher risk of biochemical recurrence for this group. To compare the NCWP-EMT and post-operative Gleason score as predictors of biochemical recurrence, two additional Cox proportional hazards models, each excluding either NCWP-EMT or post-operative Gleason score, were tested (Supplementary Table 8). The Akaike information criterion (AIC) represent the goodness of fit as well as the complexity of the model, and can be compared between models, where the lower AIC provides a better model fit. The model including post-operative Gleason had a slightly lower AIC (AIC=64.24) compared to the model including NCWP-EMT (AIC=65.61), suggesting post-operative Gleason to be a slightly better predictor of biochemical recurrence than NCWP-EMT. However, the model containing all variables, had the lowest AIC (AIC=60.15) demonstrating improved prediction of biochemical recurrence when NCWP-EMT and post-operative Gleason score were modelled together.

**Table 3:** Univariate and multivariate Cox proportional hazards analyses of biochemical recurrence

	Univariate		Multivariate – All variables(AIC = 60.15)	
Variables	Hazard ratio (95% CI)	P-values	Hazard ratio (95% CI)	P-values
Post-operative Gleason score(≤7^Δ^ and ≥8)	7.66(2.20-26.62)	0.001*	19.46(2.67-142.9)	0.003*
Pathological T-stage(≤T2c^Δ^ and ≥T3a)	6.88(2.06-23.01)	0.002*	8.27(0.89-77.15)	0.064
Pre-operative PSA(<10^Δ^ and ≥10)	2.17(0.69-7.13)	0.204	2.89(0.72-11.67)	0.14
NCWP-EMTContinuous score/100 (-4.4–5.4)	1.37(1.08-1.73)	0.009*	Low GS1.61(1.06-2.44)	Low GS0.028*
			High GS0.59(0.35-0.99)	High GS0.044*
NCWP-EMT andPost-operative Gleason score (≤7^Δ^ and ≥8)(interaction term)	-	-	0.37(0.18-0.74)	0.005*

^Δ^ Indicates the category used as a reference in each analysis.

* Indicates significant p-value.

Low GS – Hazard ratio/p-value for patients with post-operative Gleason score ≤7

High GS – Hazard ratio/p-value for patients with post-operative Gleason score ≥8

Similar findings were also visualized by using logistic regression and receiver operating characteristic (ROC) curves with the depended variable being biochemical recurrence after 5-year follow-up. The area under the curve (AUC) of the ROC-curve were the same for NCWP-EMT and post-operative Gleason score (AUC=80.9), and in combination they provided increased sensitivity and specificity (AUC=90.4) (Figure 5D). In conclusion, our results suggest that the NCWP-EMT signature could be a useful addition in prediction of biochemical recurrence in prostate cancer.

## CONCLUSIONS

The present study showed no alterations in the CWP in prostate cancer, but revealed an increased expression of NCWP and EMT markers in a subgroup of mainly *high Gleason* grade prostate cancer samples. A novel gene expression signature (NCWP-EMT) for this expression profile was presented and confirmed in several publicly available patient cohorts. *High* NCWP-EMT score was associated with reduced concentrations of the metabolites citrate and spermine both *ex vivo*, and in a clinical non-invasive setting using *in vivo* patient MRSI. The novel NCWP-EMT signature was also shown to be a predictor of biochemical recurrence and was associated with metastasis, indicating that upregulation of the NCWP and EMT is linked to more aggressive prostate cancer. The novel NCWP-EMT signature may therefore be useful for risk stratification and molecular subtyping of prostate cancer patients. The NCWP and its relation to EMT, cancer aggressiveness and tumor metabolism warrants further attention in prostate cancer studies.

## MATERIALS AND METHODS

### Patients and tissue samples

In the *main cohort*, human prostate tissue was collected from 41 localized and locally advanced prostate cancer patients. The tissue harvesting was performed on fresh-frozen prostatectomy specimens using a standardized method thoroughly described by Bertilsson et al. [29]. A total of 95 cancer tissue samples, and 34 adjacent normal tissue samples were collected (median 3, range 1-6 samples per patient). At least five years of follow-up data were successfully retrieved for 33 patients in the *main cohort*, including the date of biochemical recurrence (PSA of at least 0.2 ng/mL) and/or last negative PSA measurement. To validate the results of the *main cohort*, an additional cohort of 90 needle biopsies from 90 localized and locally advanced cancer patients were harvested and snap frozen within seconds after prostatectomy. Of these, only the samples with histopathological confirmed cancer were used as the *immunohistochemistry cohort* for this study (n=40). The patients in both cohorts received no prostate cancer treatment prior to surgery and had no detected metastasis at diagnosis. The Regional Committee of Medical and Health Research Ethics (REC), Central Norway approve both cohorts, and all patients gave written, informed consent. Validation was performed in four prostate cancer microarray datasets available through the Gene Expression Omnibus with GEO accessions GSE8218 (65 samples) [44], GSE16560 (281 samples) [45], GSE21034 (131 samples) [46], GSE46691 (545 samples) [47], and one data set from The Cancer Genome Atlas (TCGA, 497 samples) [48], in total 1519 samples (Supplementary Table 1). These datasets are collectively termed the *validation cohorts*. Biochemical recurrence was validated in the GSE21034 cohort, and metastasis in the GSE46691 cohort.

### Histopathology

In the *main cohort*, tissue slices for histopathological evaluation were cryosectioned from each tissue sample prior to HR-MAS MRS [29]. All cryosections were stained with Haematoxylin and Eosin, and the histopathological evaluations were performed according to the clinical criteria for prostate cancer, by an experienced pathologist specialized in uropathology (TV). The percentage of Gleason grades, cancer, normal glandular epithelia, and stromal tissue were reported for each sample. Reproducibility of the histopathological scoring was assessed independently by a second pathologist specialized in uropathology (ER), and the overall kappa (κ) coefficient for interobserver agreement of Gleason score was 0.66 indicating substantial agreement. The first reading was used in this study due to slight degradation of the cryosections between the readings (5 years, slides kept dry and dark). Luminal space was quantified in each sample by a color-based segmentation method (Positive Pixel Count algorithm in ImageScope v.8, Aperio Technologies) [49]. The samples in the *immunohistochemistry cohort* were formalin fixed and paraffin embedded for sectioning after HR-MAS MRS analysis, and histopathological evaluation was done according to the same protocol as the *main cohort*. In both cohorts, we investigated differences between low and high Gleason grade by sorting the tissue samples into two groups, where samples in the *low Gleason* group had a Gleason score ≤ 3+4 and samples in the *high Gleason* group had a Gleason score ≥ 4+3 (Table 1).

### HR-MAS MRS and MRSI experiments and quantification

For both the *main* and the *immunohistochemistry* cohort, proton HR-MAS MRS was acquired using a Bruker Avance DRX600 Spectrometer (Bruker Biospin, Germany) equipped with a dual ^1^H/^13^C MAS probe. Absolute quantification of the spectra was performed using LCModel [50] with a basis set of 23 metabolites, and reported in mmol/kg wet weight. Full procedure and parameters of the HR-MAS MRS acquisition and LCModel quantification have earlier been described by Giskeødegård et al. [28]. *In vivo* patient MRSI examination of the prostate, performed using a 3T system (Magnetom Trio, Siemens, Germany) prior to prostatectomy, was available on a subset of the patients in the *main cohort* (n=9). Choline, citrate, creatine and spermine were quantified using LCModel, and creatine was used as an internal standard for normalization (metabolites to creatine ratios). HR-MAS cancer samples from the same patients were spatially matched to an *in vivo* voxel (n=22). Further details on the MRSI acquisition, quantification, and spatial matching are previously described by Selnæs et al. [27].

### Gene expression, selection of genes, and controlling for confounding stroma

In the *main cohort*, gene expression analysis was performed after HR-MAS MRS on the exact same tissue sample, using an Illumina TotalPrep RNA Amplification Kit (Ambion Inc.) and an Illumina Human HT-12v4 Expression Bead Chip (Illumina), as described by Bertilsson et al. [51]. The microarray data has previously been published in Array Expression with access number: E-MTAB-1041. Genes relevant to both the WP and EMT were carefully chosen by investigating literature and publicly available pathway maps (KEGG as per March 2015) [2, 3, 5, 22]], resulting in 196 genes (Supplementary Table 2). To control for the effect of confounding stroma tissue when identifying differentially expressed genes, we used a recently published strategy of balancing the stroma content between sample groups [30]. This strategy makes it possible to separate molecular signals relevant to cancer from signals originating due to different stroma fractions between the sample groups. Briefly described, the strategy selects samples to ensure an equal average fraction of stroma tissue (according to histopathology) in each sample group termed a *balanced* differential expression analysis. In contrast, an *unbalanced* analysis is also performed to highlight differentially expressed gene due to different average fractions of stroma tissue.

### Immunohistochemistry (IHC)

In the *immunohistochemistry cohort*, IHC was performed with mouse monoclonal antibodies against Wnt5a (Sigma-Aldrich, clone 3A4, dilution 1:50), N-cadherin (Dako, clone 6G11, dilution 1:30), and E-cadherin/NCH-38 (Dako, clone NCH-38, dilution 1:100) and polyclonal rabbit antibodies against β-catenin/*CTNNB1* (PRESTIGE antibodies Sigma, dilution 1:300). The sections were counter-stained with Haematoxylin. Assessment was performed manually, and all the IHC sections were evaluated based on the average staining intensity (0-3) multiplied by the percentage of positive cancer cells (0-3), obtaining a total staining index (SI) (0-9). A SI of 0 was regarded as negative, 1-2 as weak positive staining; 3-6 as moderate, and 9 as strong positive staining (Supplementary Table 7). An experienced pathologist (AMB) validated the scoring.

### Statistical analysis

The WP and EMT genes were compared for differential expression between normal and cancer samples, and between *low* and *high Gleason* samples by t-test. All the 196 genes were considered, but to ease data analysis and presentation a subgroup 48 key and/or significantly altered genes are presented as the central genes, however, a full table of the p-values is given in Supplementary Table 2. PCA was used to further investigate and visualize the unsupervised relationship between the expressions of these central WP and EMT genes. Based on the PCA score plot, a distinct set of genes was selected to make a gene expression signature termed NCWP-EMT. The co-expression between the signature genes was investigated by Pearson's correlation, and compared to other recognized gene expression signatures in prostate cancer. The distinct gene-signature pattern from PCA and Pearson's correlation between signature genes were confirmed in the *validation cohorts*. Single sample GSEA was performed to give each of the cancer samples in the *main* and *validation cohorts* a score representing the expression of the genes in the NCWP-EMT signature [52]. The samples in each cohort were sorted into three equal sized groups of *low, intermediate*, and *high* NCWP-EMT signature scores, where the *high score* group had the highest pathway activity. Features associated with NCWP-EMT were investigated by Gene Ontology (GO) using the Database for Annotation and Visualization and Integrated Discovery (DAVID). Biochemical recurrence free survival for the NCWP-EMT score groups were plotted by Kaplan-Meier curves and tested by log-rank test in the *main* and GSE21034 cohort, where for the individual patient's highest NCWP-EMT score was used in the *main cohort*. The association between NCWP-EMT and metastasis in the GSE46691 cohort was tested using a contingency table and chi-squared test. Univariate and multivariate cox proportional hazards statistics were used to investigate the role of the NCWP-EMT signature in prediction of biochemical recurrence. Prior to analysis, post-operative Gleason score, pathological T-stage and pre-operative PSA were dichotomized (Table 3), and together with the continuous NCWP-EMT signature score selected for multivariate analysis. Biochemical recurrence at five-year follow-up was selected to plot ROC curves of NCWP-EMT score, post-operative Gleason score and both combined. Linear mixed model (LMM) was used to account for multiple samples per patient, when investigating the relationship between NCWP-EMT score groups and metabolite concentrations. The analyses were repeated with additional adjustment for Gleason grade, and tissue heterogeneity including the proportion of cancer, benign epithelium, stroma and luminal space in the individual tissue sample. The *immunohistochemistry cohort* consisted of one sample per patient, and t-test was used to investigate the association between IHC and metabolite concentrations. Prior to analysis, all metabolite values were log transformed to obtain normalized residuals, and p-values were corrected for multiple testing using Benjamini-Hochberg false discovery rate. P-values <0.05 were considered significant. The statistical analyses were performed in R (version 3.2.0, R Foundation for Statistical Computing).

## SUPPLEMENTARY FIGURES AND TABLES


