# Supplementary material for: A novel non-canonical Wnt signature for prostate cancer aggressiveness
Source: Oncotarget. 2016 Dec 24;8(6):9572–86. doi: 10.18632/oncotarget.14161 (PMC5354754; doi:10.18632/oncotarget.14161)
Supplement: Supplementary file 1 [file oncotarget-08-9572-s001.pdf]

# A novel non-canonical Wnt signature for prostate cancer aggressiveness

## SUPPLEMENTARY FIGURES AND TABLES

**A** **The *main cohort***

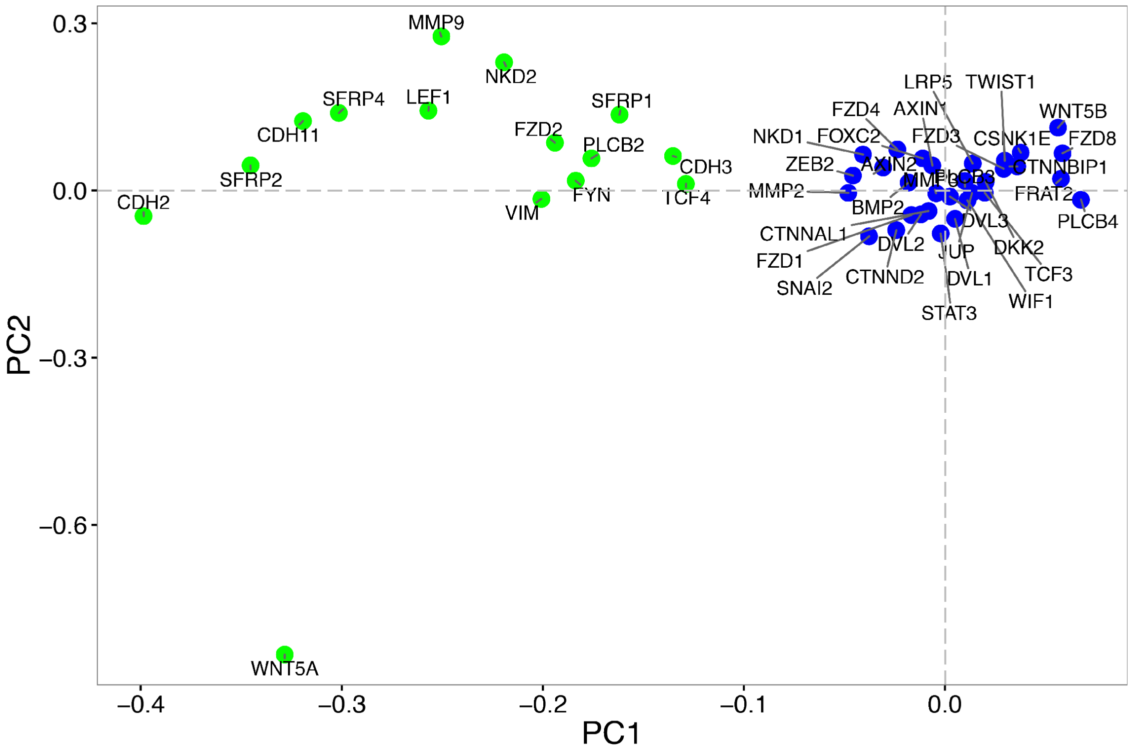


(*Continued*)

Figure S1

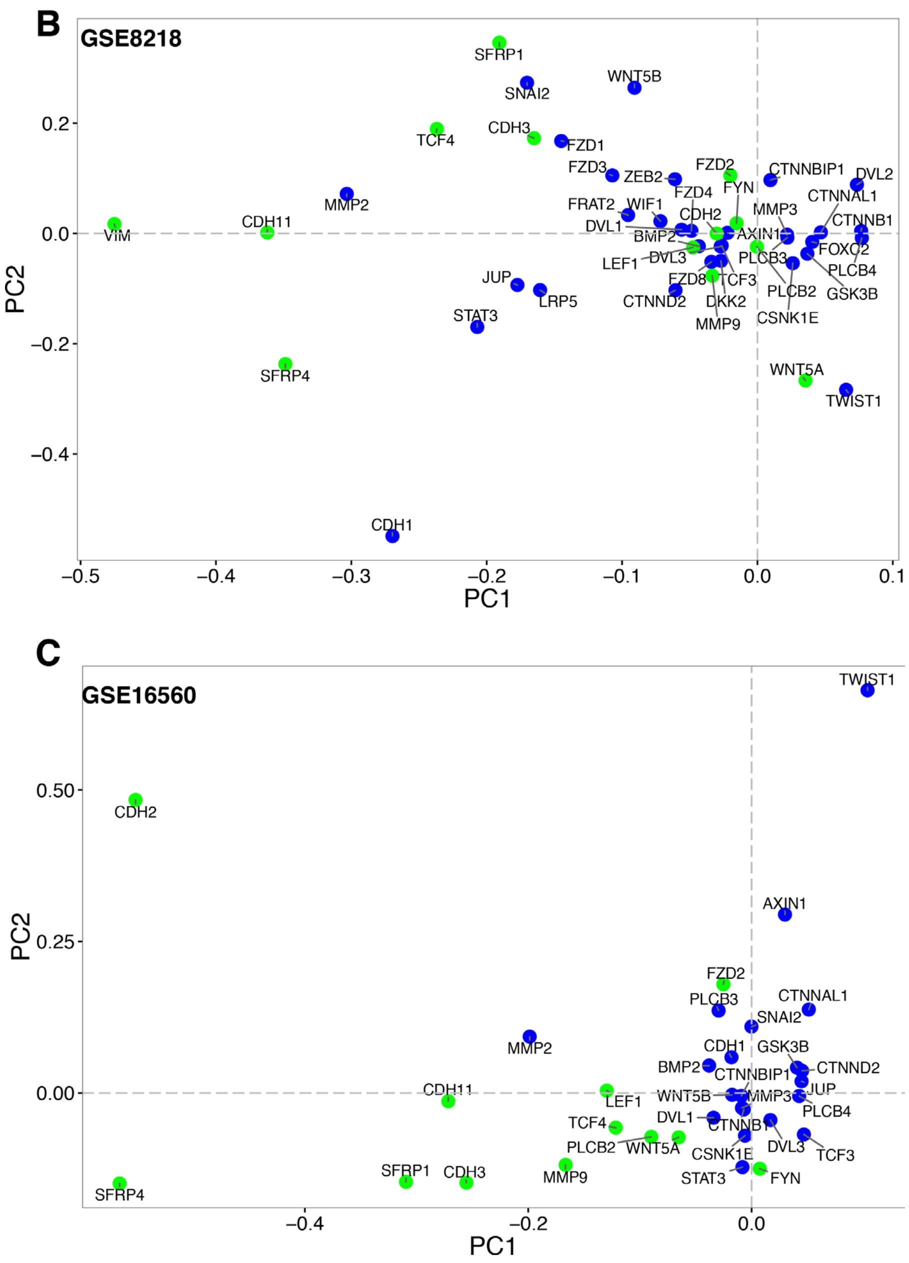


(*Continued*)

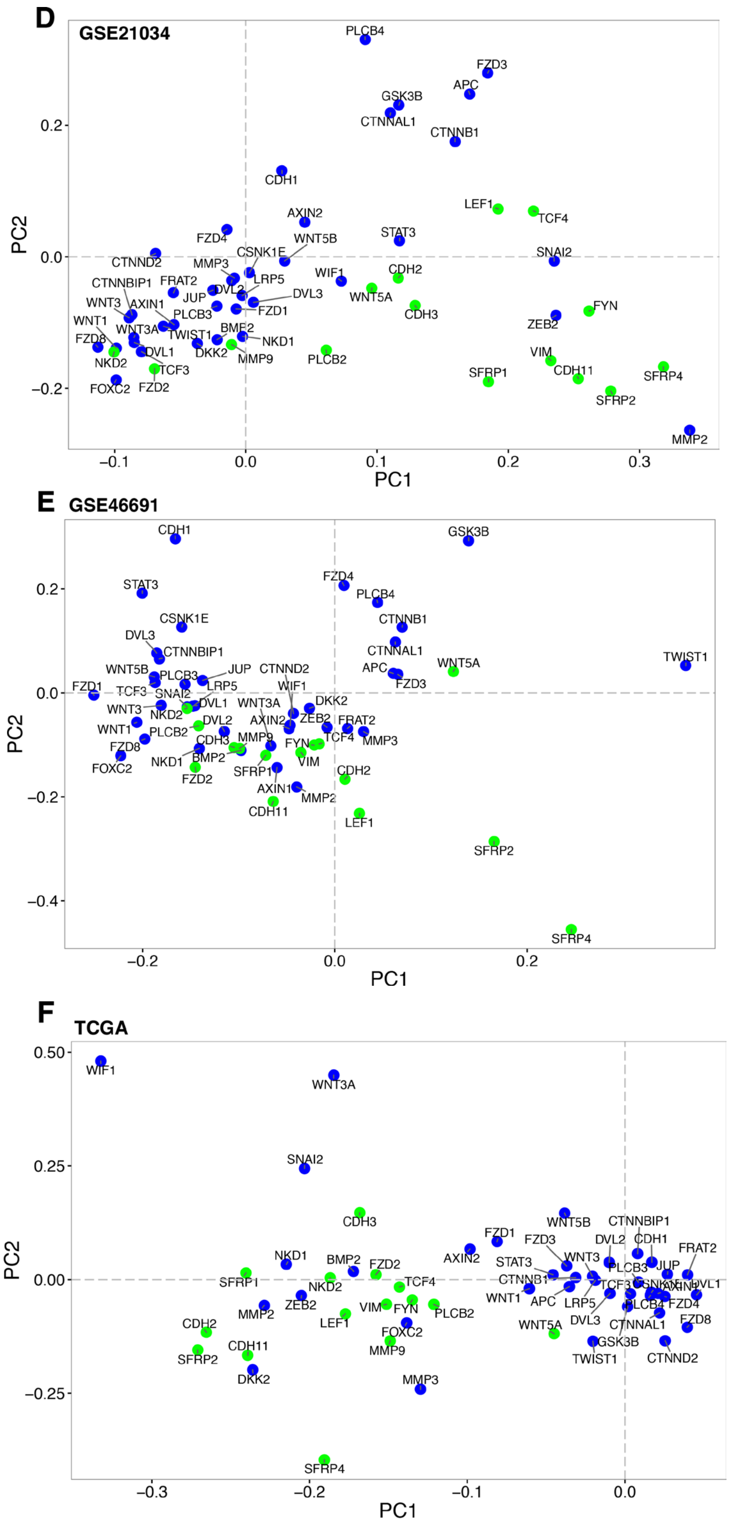


**Supplementary Figure 1: A-F.** PCA plots of the central WNT-EMT genes in the main cohort (A) and validation cohorts **D-E.** Genes in the NCWP-EMT signature is marked in green.

(*Continued*)

G

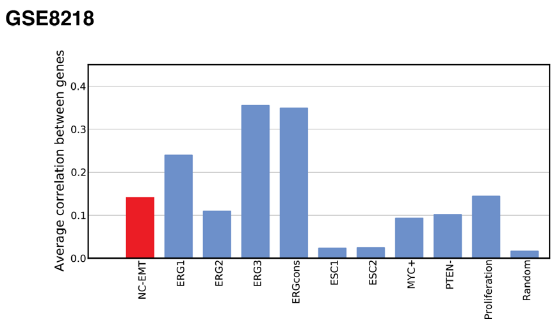


H

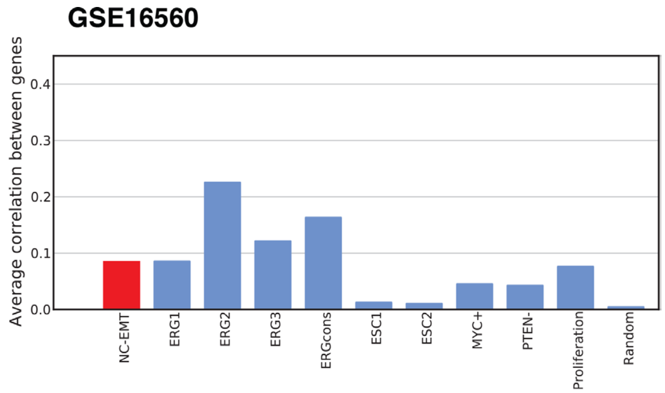


I

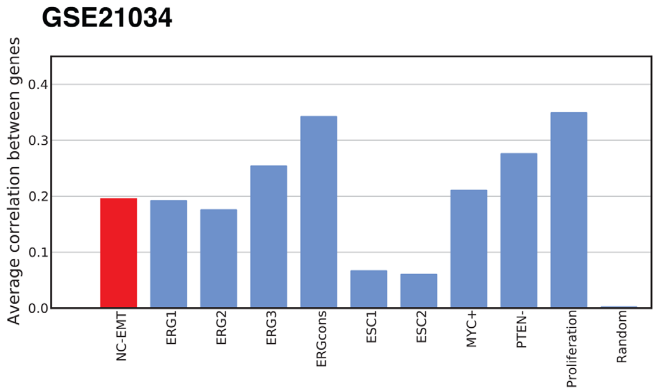


J

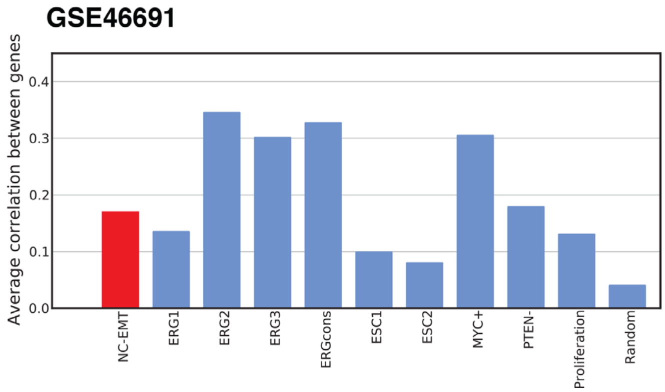


**Supplementary Figure 1** ***(Continued)*****: G-J.** NCWP-EMT gene signature co-expression correlations (red), compared to other gene signatures (blue). (see Supplementary Table S1 for references on the validation cohorts.)

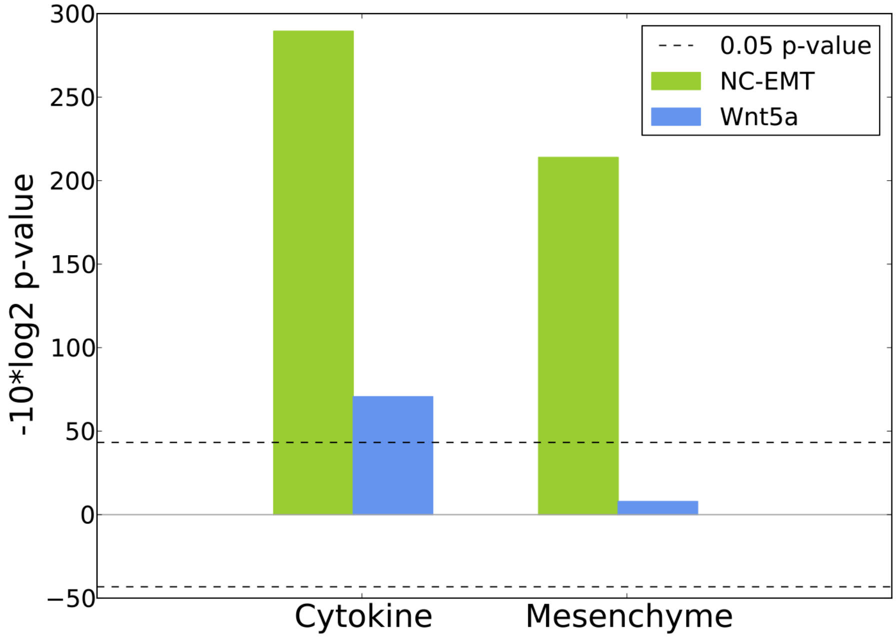


**Supplementary Figure 2: P-values plot of NCWP-EMT (green) and Wnt5a (blue) coexpression with cytokine and mesenchyme gene expression signatures.**

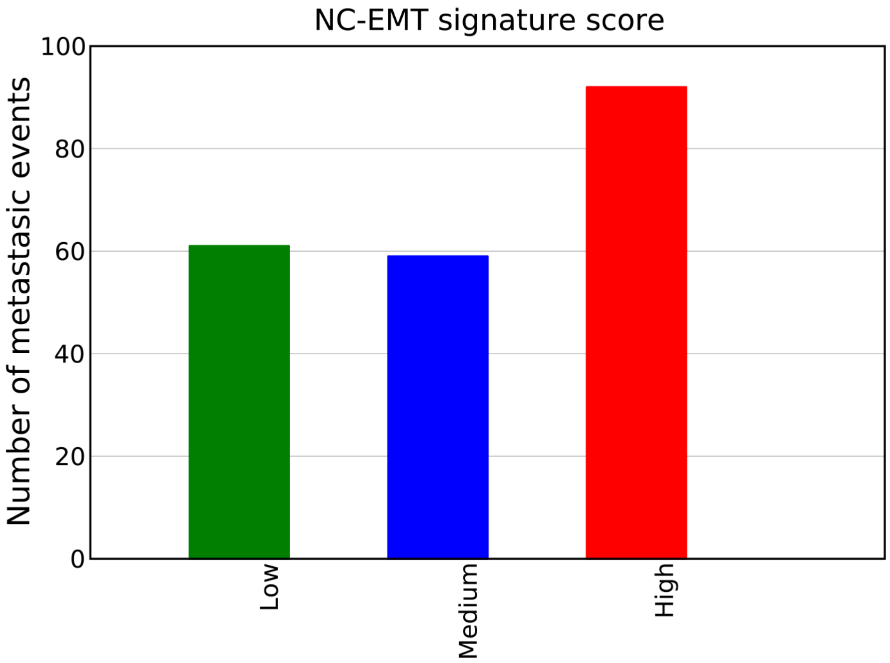


**Supplementary Figure 3: The number of metastatic events was significantly higher in high NCWP-EMT score patients (red) compared to low (green) and intermediate (blue) score patients, Chi-square test p-value 0.00029.**

**Supplementary Table 1: Overview of the validation cohorts used in the study**

| GEO accession | Total number of PCa samples | Low Gleason <= 7 | High Gleason >= 8 | Wnt-genes present in data (total=53) | NC-EMT genes present in data (total=15) | Type of follow up data (events) | Reference |
|---|---|---|---|---|---|---|---|
| GSE8218 | 65 | 54 | 11 | 45 | 13 | survival | (1) |
| GSE16560 | 281 | 200 | 81 | 33 | 12 | survival | (2) |
| GSE21034 | 131 | 120 | 11 | 53 | 15 | recurrence (27) | (3) |
| GSE46691 | 545 | 334 | 211 | 53 | 15 | metastasis (212) | (4) |
| TCGA | 497 | 297 | 200 | 53 | 15 | No data | (5) |

1. Wang Y, Xia XQ, Jia Z, Sawyers A, Yao H, Wang-Rodriquez J, et al. In silico estimates of tissue components in surgical samples based on expression profiling data. Cancer Res. 2010;70:6448-55.
2. Sboner A, Demichelis F, Calza S, Pawitan Y, Setlur SR, Hoshida Y, et al. Molecular sampling of prostate cancer: a dilemma for predicting disease progression. BMC medical genomics. 2010;3:8.
3. Taylor BS, Schultz N, Hieronymus H, Gopalan A, Xiao Y, Carver BS, et al. Integrative genomic profiling of human prostate cancer. Cancer cell. 2010;18:11-22.
4. Erho N, Crisan A, Vergara IA, Mitra AP, Ghadessi M, Buerki C, et al. Discovery and Validation of a Prostate Cancer Genomic Classifier that Predicts Early Metastasis Following Radical Prostatectomy. PLoS One. 2013;8.
5. The Cancer Genome Atlas (TCGA) [Available from: http://cancergenome.nih.gov/].

**Supplementary Table 2: Overview of all the selected genes used for analysis of the Wnt Pathway, and the p-values for alterations in expression between cancer and normal, and high and low Gleason samples.** In addition pathway classification and relevance in prostate cancer based on previous literature is noted, as well as our own classification of the genes in relation to prostate cancer**.**

See Supplementary File 1

**Supplementary Table 3: Most significant GO-terms for top 1000 differentially expressed genes between samples with high and low NCWP-EMT GSEA score, and between samples with high and low expression levels of Wnt5a**

| Source | Term | Benjamini corrected p-value |
|---|---|---|
| **NC-EMT** | | |
| **Terms related to cell surface and extracellular functions (Mesenchymal):** | | |
| SP_PIR_KEYWORDS | signal | 5.3e-27 |
| UP_SEQ_FEATURE | signal peptide | 5.7e-26 |
| SP_PIR_KEYWORDS | glycoprotein | 3.3e-20 |
| UP_SEQ_FEATURE | glycosylation site: N-linked(GlcNAc?) | 9.0e-16 |
| GOTERM_CC_FAT | extracellular region part | 6.4e-16 |
| UP_SEQ_FEATURE | topological domain: Extracellular | 9.0e-12 |
| GOTERM_CC_FAT | proteinaceous extracellular matrix | 7.2e-13 |
| GOTERM_BP_FAT | biological adhesion | 1.2e-11 |
| GOTERM_BP_FAT | cell adhesion | 1.5e-11 |
| SP_PIR_KEYWORDS | Secreted | 8.9e-11 |
| GOTERM_CC_FAT | extracellular matrix | 1.2e-11 |
| GOTERM_CC_FAT | extracellular region | 3.4e-10 |
| GOTERM_CC_FAT | plasma membrane part | 1.4e-10 |
| GOTERM_CC_FAT | integral to plasma membrane | 3.4e-10 |
| GOTERM_CC_FAT | plasma membrane | 2.9e-10 |
| GOTERM_CC_FAT | intrinsic to plasma membrane | 4.2e-10 |
| GOTERM_CC_FAT | extracellular space | 2.2e-6 |
| **Terms related immune response and inflammation (Cytokine):** | | |
| GOTERM_BP_FAT | positive regulation of immune system process | 7.0e-15 |
| GOTERM_BP_FAT | positive regulation of immune response | 4.9e-11 |
| GOTERM_BP_FAT | response to wounding | 7.5e-9 |
| GOTERM_BP_FAT | inflammatory response | 8.1e-9 |
| GOTERM_BP_FAT | defense response | 9.3e-9 |
| GOTERM_BP_FAT | positive regulation of cell activation | 1.4e-9 |
| GOTERM_BP_FAT | regulation of lymphocyte activation | 7.8e-9 |
| GOTERM_BP_FAT | regulation of T cell activation | 1.5e-8 |
| GOTERM_BP_FAT | regulation of leukocyte activation | 2.3e-8 |
| GOTERM_BP_FAT | positive regulation of response to stimulus | 2.0e-7 |
| GOTERM_BP_FAT | activation of immune response | 1.3e-5 |
| GOTERM_BP_FAT | immune effector process | 5.2e-5 |
| **WNT5A** | | |
| SP_PIR_KEYWORDS | SH2 domain | 1.2e-2 |
| GOTERM_MF_FAT | kinase binding | 8.6e-2 |
| SP_PIR_KEYWORDS | membrane | 2.0e-2 |
| SP_PIR_KEYWORDS | transmembrane | 6.2e-2 |
| BIOCARTA | T Helper Cell Surface Molecules | 5.8e-2 |
| SP_PIR_KEYWORDS | Ehler Danlos syndrome | 5.6e-2 |

**Supplementary Table 4: Metabolite concentration and alterations between low, intermediate and high NC-EMT signature score, for the main cohort**

| | Metabolite concentration (mmol/kg wet weight) | | | P-values [a] | | |
|---|---|---|---|---|---|---|
| **Signature score** | **Low** | **Int** | **High** | **High/Low** | **High/Int** | **Int/Low** |
| **Metabolite** | **Median (IQR) (n=32)** | **Median (IQR) (n=31)** | **Median (IQR) (n=32)** | | | |
| Alanine | 2.23 (1.68-2.82) | 2.46 (1.87-3.20) | 2.08 (1.65-2.60) | 0.407 | 0.228 | 0.792 |
| Choline | 1.07 (0.68-1.42) | 1.03 (0.69-1.92) | 1.06 (0.66-1.65) | 0.806 | 0.511 | 0.792 |
| Citrate | 7.31 (5.57-11.56) | 6.38 (4.56-11.58) | 3.55 (2.08-7.25) | 3.38E-04* | 0.018* | 0.282 |
| Creatine | 1.93 (1.39-2.71) | 2.32 (2.01-2.67) | 1.98 (1.61-2.50) | 0.684 | 0.259 | 0.592 |
| Ethanolamine | 0.00 (0.00-0.29) | 0.00 (0.00-0.17) | 0.00 (0.00-0.19) | 0.881 | 0.884 | 0.938 |
| Glucose | 0.09 (0.00-0.52) | 0.00 (0.00-0.36) | 0.00 (0.00-0.25) | 0.449 | 0.884 | 0.658 |
| Glutamate | 4.68 (3.20-6.67) | 5.77 (3.85-7.52) | 5.24 (4.21-7.51) | 0.974 | 0.905 | 0.993 |
| Glutamine | 2.75 (2.10-3.55) | 2.76 (2.27-3.86) | 2.80 (2.53-3.49) | 0.974 | 0.884 | 0.938 |
| GPC | 0.98 (0.53-1.36) | 0.44 (0.73-1.16) | 0.74 (0.50-1.06) | 0.407 | 0.578 | 0.754 |
| GPEA | 0.00 (0.00-0.68) | 0.09 (0.00-0.53) | 0.10 (0.00-0.58) | 0.958 | 0.884 | 0.938 |
| Glycine | 2.38 (1.57-3.04) | 2.48 (1.89-3.43) | 2.55 (1.91-3.63) | 0.881 | 0.884 | 0.754 |
| Isoleucine | 0.12 (0.00-0.20) | 0.17 (0.12-0.29) | 0.19 (0.10-0.32) | 0.163 | 0.884 | 0.132 |
| Lactate | 16.74 (13.66-22.57) | 21.86 (15.62-26.41) | 20.31 (16.47-25.26) | 0.974 | 0.511 | 0.425 |
| Leucine | 0.42 (0.21-0.58) | 0.49 (0.36-0.65) | 0.57 (0.33-0.93) | 0.684 | 0.884 | 0.792 |
| Myo-inositol | 8.17 (6.46-10.21) | 9.83 (7.43-12.87) | 9.55 (8.36-11.65) | 0.834 | 0.497 | 0.131 |
| Phosphocholine | 0.55 (0.28-1.04) | 0.87 (0.54-1.17) | 0.74 (0.48-1.32) | 0.806 | 0.689 | 0.282 |
| PEA | 2.18 (1.25-2.89) | 2.92 (2.23-4.00) | 2.88 (2.20-3.79) | 0.159 | 0.511 | 2.14E-03* |
| Putrescine | 0.04 (0.00-0.35) | 0.08 (0.00-0.20) | 0.00 (0.00-0.53) | 0.601 | 0.884 | 0.754 |
| Scyllo-inositol | 0.41 (0.32-0.59) | 0.44 (0.32-0.64) | 0.45 (0.38-0.62) | 0.589 | 0.327 | 0.792 |
| Spermine | 1.55 (1.02-2.36) | 1.23 (0.67-2.27) | 0.75 (0.39-1.43) | 3.38E-04* | 0.028* | 0.113 |
| Succinate | 0.64 (0.42-0.88) | 0.61 (0.49-0.94) | 0.60 (0.45-0.71) | 0.589 | 0.511 | 0.965 |
| Taurine | 3.84 (2.84-4.94) | 4.05 (4.62-6.96) | 5.76 (4.03-7.28) | 0.131 | 0.884 | 0.022* |
| Valine | 0.32 (0.20-0.46) | 0.38 (0.24-0.56) | 0.41 (0.28-0.59) | 0.806 | 0.689 | 0.938 |

*ex vivo*.

[a] P-values from Linear mixed model adjusted or multiple samples per patient, corrected for multiple testing by the Benjamini and Hochberg procedure.

* Indicates signficant p-values

Abbreviations: Int - Intermediate, IQR - Interquartile range, GPC - Glycerophosphocholine, GPEA - Glycerophosphoethanolamine, PEA - Phosphoethanolamine.

**Supplementary Table 5: P-values for metabolite alteration between low and high NC-EMT samples, adjusting for Gleason risk and tissue heterogeneity**

| | P-values [a] | | | | | |
|---|---|---|---|---|---|---|
| **Adjusted for:** | **Patient** | **Patient, Gleason risk** | **Patient, Stroma** | **Patient, Cancer** | **Patient, Benign epithelium** | **Patient, Luminal space** |
| Alanine | 0.407 | 0.352 | 0.623 | 0.509 | 0.329 | 0.480 |
| Choline | 0.806 | 0.839 | 0.847 | 0.837 | 0.818 | 0.735 |
| Citrate | 3.38E-04* | 2.42E-03* | 4.20E-04* | 2.01E-04* | 8.61E-04* | 2.81E-03 * |
| Creatine | 0.684 | 0.855 | 0.650 | 0.671 | 0.805 | 0.735 |
| Ethanolamine | 0.881 | 0.958 | 0.950 | 0.837 | 0.867 | 0.839 |
| Glucose | 0.449 | 0.657 | 0.109 | 0.176 | 0.515 | 0.417 |
| Glutamate | 0.974 | 0.839 | 0.650 | 0.916 | 0.856 | 0.965 |
| Glutamine | 0.974 | 0.839 | 0.650 | 0.837 | 0.867 | 0.903 |
| GPC | 0.407 | 0.303 | 0.623 | 0.509 | 0.249 | 0.578 |
| GPEA | 0.958 | 0.958 | 0.734 | 0.837 | 0.933 | 0.965 |
| Glycine | 0.881 | 0.955 | 0.623 | 0.671 | 0.981 | 0.965 |
| Isoleucine | 0.163 | 0.143 | 0.119 | 0.176 | 0.109 | 0.239 |
| Lactate | 0.974 | 0.958 | 0.650 | 0.837 | 0.933 | 0.965 |
| Leucine | 0.684 | 0.958 | 0.542 | 0.587 | 0.818 | 0.735 |
| Myo-inositol | 0.834 | 0.839 | 0.650 | 0.787 | 0.832 | 0.735 |
| Phosphocholine | 0.806 | 0.958 | 0.623 | 0.587 | 0.867 | 0.735 |
| PEA | 0.159 | 0.143 | 0.109 | 0.127 | 0.221 | 0.246 |
| Putrescine | 0.601 | 0.397 | 0.623 | 0.587 | 0.805 | 0.691 |
| Scyllo-inositol | 0.589 | 0.566 | 0.650 | 0.587 | 0.515 | 0.735 |
| Spermine | 3.38E-04* | 1.62E-03* | 4.20E-04* | 2.01E-04* | 8.61E-04* | 2.81E-03* |
| Succinate | 0.589 | 0.414 | 0.767 | 0.658 | 0.515 | 0.691 |
| Taurine | 0.131 | 0.143 | 0.119 | 0.127 | 0.092 | 0.161 |
| Valine | 0.806 | 0.958 | 0.623 | 0.671 | 0.828 | 0.843 |

[a] P-values from Linear mixed modell, corrected for multiple testing by the Benjamini and Hochberg procedure.
* Indicates signficant p-values
Abbreviations: GPC - Glycerophosphocholine, GPEA - Glycerophosphoethanolamine, PEA - Phosphoethanolamine.

**Supplementary Table 6: There were no metabolic differences between low, intermediate and high WNT5A gene expression in the main cohort or between low/moderate and high Wnt5a IHC expression in the validation cohort**

| | Main cohort P-values [a] | | | Validation cohort P-values [b] |
|---|---|---|---|---|
| **WNT5A expression/ Metabolite** | **Low/Int** | **Low/High** | **Int/High** | **IHC: Low/High** |
| Alanine | 0.502 | 0.710 | 0.990 | 0.670 |
| Choline | 0.836 | 0.826 | 0.990 | 0.926 |
| Citrate | 0.715 | 0.826 | 0.892 | 0.623 |
| Creatine | 0.882 | 0.826 | 0.892 | 0.406 |
| Ethanolamine | 0.987 | 0.240 | 0.190 | 0.175 |
| Glucose | 0.836 | 0.882 | 0.990 | 0.396 |
| Glutamate | 0.836 | 0.882 | 0.892 | 0.755 |
| Glutamine | 0.836 | 0.882 | 0.990 | 0.672 |
| GPC | 0.715 | 0.882 | 0.990 | 0.433 |
| GPEA | 0.744 | 0.826 | 0.990 | 0.666 |
| Glycine | 0.638 | 0.882 | 0.892 | 0.746 |
| Isoleucine | 0.855 | 0.882 | 0.892 | 0.367 |
| Lactate | 0.684 | 0.882 | 0.892 | 0.682 |
| Leucine | 0.744 | 0.826 | 0.892 | 0.117 |
| Myo-inositol | 0.684 | 0.826 | 0.990 | 0.603 |
| Phosphocholine | 0.638 | 0.882 | 0.892 | 0.628 |
| PEA | 0.568 | 0.826 | 0.892 | 0.610 |
| Putrescine | 0.502 | 0.710 | 0.990 | 0.423 |
| Scyllo-inositol | 0.684 | 0.826 | 0.990 | 0.765 |
| Spermine | 0.836 | 0.918 | 0.990 | 0.619 |
| Succinate | 0.638 | 0.826 | 0.990 | 0.498 |
| Taurine | 0.502 | 0.826 | 0.892 | 0.430 |
| Valine | 0.684 | 0.826 | 0.990 | 0.313 |

[a] P-values from Linear mixed model adjusted or multiple samples per patient, corrected for multiple testing by the Benjamini and Hochberg procedure.
[b] P-values from independent samples t-test. Not corrected for multiple testing
Abbreviations: Int - Intermediate, GPC - Glycerophosphocholine,
GPEA - Glycerophosphoethanolamine, PEA - Phosphoethanolamine.

**Supplementary Table 7: Immunohistochemistry scoring for staining index (SI)**

| Score | 0 | 1 | 2 | 3 |
|---|---|---|---|---|
| Staining intensity | No detectable staining | Weak staining | Moderate staining | Strong staining |
| Percentage of positive cells | 0% | 1-10% | 11-50% | >50% |
| Staining index (SI) | 0 | 1,2 | 3,4,6 | 9 |
| Staining classification | Negative | Weak | Moderate | Strong |

Staining index (SI) is obtained by multiplying the scores of staining intensity and percentage of positive cells.

**Supplementary Table 8: Multivariate cox proportional hazards analyses of biochemical recurrence**

| | NCWP-EMT model (AIC = 65.61) | | Post-operative Gleason score model (AIC = 64.24) | |
|---|---|---|---|---|
| **Variables** | **Hazard ratio (95% CI)** | **P-value** | **Hazard ratio (95% CI)** | **P-value** |
| **Post-operative Gleason score** (≤7* and ≥8) | - | - | 5.42 (0.86-34.11) | 0.072 |
| **Pathological T-stage** (≤T2c* and ≥T3a) | 3.79 (0.91-1.63) | 0.088 | 2.12 (0.35-12.77) | 0.41 |
| **Preoperative PSA** ≤10* and ≥10 | 2.14 (9.82-17.5) | 0.22 | 2.47 (0.69-8.85) | 0.17 |
| **NCWP-EMT** Continuous score/100 (-4.4-5.4) | 1.22 (0.91-1.63) | 0.188 | - | - |

* Indicates the group used as a reference in each analysis.
